# News and Coming Events

**Published:** 1909-06-12

**Authors:** 


### News and Coming Events.

Lady Midleton has consented to open, on June 18, the convalescent home at Worthing now in process of erection as an adjunct to the Royal Surrey County Hospital, which is largely due to Mr. G. Prentiss Shepherd's donation of £10,000 towards its erection and endowment. A further sum of £1,000 has been received by this hospital for the provision of an x-ray department from Miss Helen Crooke as a memorial to her late father, Mr. Frederick Augustine Crooke, who was Mayor of Guildford in 1877.

At the University of London the following elections to studentships were announced on June 5: Miss Mary Taylor Fraser, B.Sc., to the Lindley Studentship, of £100, open to students qualified to undertake research in physiology, and tenable in the Physiological Laboratory of the University.—Mr. G. Roche Lynch, St. Mary's Hospital, to the University Studentship in Physiology, of £50, open to students qualified to undertake research in physiology, and tenable for one year in the Physiological Laboratory of the University or of a school of the University.

The case of Fergusson v. Malvern Urban District Council recently reached its final stage of appeal in the House of Lords. It will be remembered that Dr. J. C. Fergusson, lessee and proprietor of a hydropathic establishment at Malvern, alleged that the water, which led to an outbreak of enteric fever in his institution, became polluted by the negligence of the defendants, who allowed a well from which he, to their knowledge, drew a part of his supply, to become contaminated with sewage by percolation. He claimed the right to take this water in future free from pollution, and asked for damages based on the compensation he had had to pay to those who became ill while in his house, and for loss of business and injury to his reputation. The action was originally tried before Mr. Justice Lawrance and a special jury, and a verdict for £7,500 was returned for the plaintiff. The Court of Appeal set aside that verdict, since the plaintiff had failed to establish any proprietary right in this water supply, and, therefore, took it at his own risk. The Lord Chancellor upheld the decision of the Court of Appeal in this case, which, he said, presented many difficulties. The plaintiff, to succeed, must show that he had some right to take the water from this spring, and he failed to show any such right. His Lordship failed to see how, if a new trial were ordered, the plaintiff could succeed, and he moved that the appeal should be dismissed, with costs. The other Lords concurred with some reluctance.

At a recent meeting of the Metropolitan Asylums Board a report was read from the Hospitals Committee with reference to a letter from Dr. H. R. Kenwood, Professor of Hygiene at University College, London, inquiring on behalf of his class in Public Health whether "the existing conditions under which they are at present signed up for the three months' attendance upon a fever hospital which is required by the regulations for the diploma" can be modified. The committee, after considering the matter very carefully, recommended: "That, subject to the sanction of the Local Government Board, the existing facilities for the study of hospital administration afforded to candidates for the Diploma in Public Health be supplemented by the institution at two of the hospitals, as an experiment, of classes for the instruction of candidates in hospital administration without requiring them to enter into residence, and that the Local Government Board be furnished with a copy of the report on the subject by the medical officer for the general purposes"; and this was agreed to without comment.

Mrs. Annie Jane Holborn, of Campden Hill, W., who died in April last, amongst many other charitable bequests, left £5,000 to St. Bartholomew's Hospital; and £500 each to the Kensington Dispensary and Children's Hospital for a "Kensington Chapel" cot, and to the Home for Confirmed Invalids, Highbury.

A Conference on "The Medical Inspection and Treatment of School Children" will be held at the Incorporated Institute of Hygiene, 34 Devonshire Street, Harley Street, W., on June 21. It is intended to consider the problem of "How to Deal with Defective Children," and a number of prominent medical men have arranged to take part in the discussion Sir William Bennett, F.R.C.S., K.C.V.O., will preside.

At the last meeting of the Metropolitan Asylums Board the receipt of a letter was reported from the Local Government Board stating that they have no objection to the experimental establishment of classes at two of the managers' hospitals for the instruction of candidates for the diploma in Public Health in hospital administration, provided that the proposal could be carried out without cost to the rates.

The Public Health Department of the City of London has issued a circular, for the guidance of persons dealing in meat, drawn up by its Medical Officer of Health, Dr. Collingridge, and Mr. King, Veterinary Inspector to the Corporation, which describes the indications of tuberculosis both in the carcass and in the living animal. Copies can be obtained on application at the Public Health Department at the Guildhall.

The Eleventh Annual Conference of the American Hospital Association is announced for September 21 to 24 inclusive. The Conference will be held at the New Willard Hotel, Washington, D.C., and a preliminary programme has already been issued, giving the names of members of the various committees of the Association, and a list of the addresses, papers, reports and exhibitions which will follow the Address of Welcome and the Presidential Address on the morning of Tuesday, September 21. Particulars of hotel tariffs and railway facilities are also given. The final programme will be issued early in September. Many topics of the deepest interest to all connected in any way with hospitals and hospital administration will be dealt with, and several social problems arising out of medical charity or relating to it will form the subject of papers and discussions.

### LEPERS AT THE AGRICULTURAL HALL.

A distinctive feature of the Exhibition—"Africa and the East"—opened by the Archbishop of Canterbury on Tuesday, June 8, at the Agricultural Hall, is "The Leper Court." Here the work of the Mission to Lepers in India and the East is illustrated by means of models of Indian and Chinese asylums, specially prepared photographs, curios, etc. The Mission is now at work in seventy-eight settlements or asylums, containing fully 8,000 lepers; and 500 untainted children are in the Homes connected with the Society. The exhibit is being arranged by Mr. John Jackson, F.R.G.S., who will also give addresses daily on the tour among the leper settlements of India, China and Japan, from which he has recently returned. Other speakers will be Mr. T. A. Bailey and Mr. C. Douglas Green.

The late Mr. Claudius Galen Wheelhouse, F.R.C.S., D.Sc., LL.D., of Filey, Yorks, whose recent death at the age of 82 we recorded a few weeks ago, left estate valued at £18,590 gross, with net personalty £17,481.

The annual dinner of the Indian Medical Service was held on Thursday, June 10, at the Gaiety Restaurant, when General Sir O'Moore Creagh was the guest of the evening and Sir George Birdwood was in the chair.

Dr. Henry Kay Ramsden, of Sloane Street, Chelsea, died last week after a long illness at the age of forty-two. After studying at Owens College Dr. Ramsden obtained the M.B., Ch.B. degree at Victoria University in 1891, and held various resident and other appointments in London, Guernsey, and Queensland. He had also been a civil surgeon with the South African Field Force, and was a surgeon captain in the 6th London Rifles.

The Army Council has approved arrangements for the training at military hospitals of the matrons on the rolls of Territorial general hospitals. Each of the latter is allowed two matrons, all liable to be called up for training for seven days in alternate years. The hospitals appointed for the training are the Royal Victoria Hospital, Netley; Queen Alexandra Military Hospital, Millbank; Cambridge Hospital, Aldershot; Connaught Hospital, Aldershot; Military Hospital, Devonport; Royal Herbert Hospital, Woolwich; Alexandra Hospital, Cosham; and Military Hospital, Colchester.

Mr. H. Cosmo O. Bonsor, treasurer of Guy's Hospital, in the annual report states with satisfaction that, while the existing debt was paid off in 1907, bequests to the extent of £8,617 more than made up the deficiency in ordinary income. Moreover, the current cost of the new out-patient department now in progress has been met from the fund to which an anonymous donor and the council of King Edward's Hospital Fund have already contributed the greater portion of the sum needed. The number of beds available for use has been increased from 608 to 620. The average number of in-patients was 529, and the total number 8,059. The total number of new out-patients was 132,288, with 446,662 separate attendances. The number of deaths in the hospital was 701, of which 86 were due to accidents. The income totalled £71,488 and the ordinary expenditure £65,775, the balance being placed to the credit of capital. The Re-endowment Fund, instituted in 1896, now amounts to £281,421, of which £6,003 was added last year.

The annual meeting of the National Association for the Prevention of Consumption and Other Forms of Tuberculosis was held on June 4 at the Tuberculosis Exhibition, which has been arranged by the association at the Whitechapel Art Gallery. Lord Balfour of Burleigh, chairman of council, presided, and was supported by Sir Constantine Holman, Dr. A. Ransome, of Bournemouth, Dr. Theodore Williams, Dr. R. W. Philip, of Edinburgh, and Miss Broadbent. The chairman, in moving the adoption of the report, dwelt with approbation upon the signs which are now evident of the gradual awakening of the Government and the public to the gravity of the tuberculosis problem, and the need for co-operation between all classes in the fight against consumption. He stated that five thousand people passed the turnstiles on the opening day of the exhibition, and the whole available stock of literature had been distributed with the best possible chance of going where it could be of greatest service. A considerable proportion of the visitors obviously came with the object of learning and profiting by what they saw and heard.

The annual festival dinner in aid of King's College Hospital, London, will be held in the Victoria Hall of the Hotel Cecil on Monday, June 21, at 7 o'clock for 7.30 p.m. precisely. The chair will be taken by Lord Northcote.

The Right Hon. R. B. Haldane, M.P., F.R.S., Secretary of State for War, will visit University College, London, on Friday, June 18, at 4.45 p.m., for the purpose of opening the new Institute of Physiology. Academic dress will be worn.

A conference of Charity Organisation Societies is now being held at Malvern and Worcester. The proceedings include a Reception, and papers and discussions upon topics related to the organisation, co-operation, and administration of charitable institutions.

Mr. J. Hampton Hale presided on June 2 at the quarterly Court of the Governors of the London Hospital. The House Committee reported upon the administration of the spinal anæsthetic stovaine. In their opinion the shock to the patient is far less than the shock from ether or chloroform, and stovaine is far safer for anæsthetising patients with cardiac disorders. The committee view with apprehension the proposed new taxes on alcohol and rectified spirit, which it is estimated will increase the dispensary account of the hospital by £200 a year.

In his last monthly report Dr. Collingridge, Medical Officer of Health for the City, reports upon a further series of 32 samples of milk taken at the City railway stations and submitted for examination to Dr. Klein, F.R.S., of St. Bartholomew's Hospital. Of these 15 per cent. contained an appreciable amount of dirt, 40 per cent. had pathogenic bacteria of some kind, and of these latter 12.5 per cent. produced in guinea-pigs definite tuberculosis with the typical tubercle bacilli. Only 43.7 per cent. could be classed as clean and pure. Of a number of guinea-pigs inoculated with some sediment from the samples, one died with severe inflammation of the lungs and another with extensive pseudo-tuberculosis. The percentage of tubercle-infected milk was the largest ever recorded in the City, a rise from 7.7 to 12.5. A deplorable state of affairs was revealed in the subsequent tracing of the infected samples to their farms of origin, which exemplified the gross recklessness and absolute want of precaution with which the average farmer trades in milk. Dr. Collingridge strongly condemns the practice of mixing milk from churns on the great railway termini platforms.

### LIVINGSTONE COLLEGE.

Commemoration Day was celebrated at Livingstone College on May 26. Many distinguished visitors travelled to the College at Leyton to take part in the proceedings. The Master of Trinity College, Cambridge (Dr. Butler) presided, and the Principal (Dr. C. F. Harford) described the progress which has taken place since Professor Macalister's Inaugural Address fifteen years ago. He pointed out the change which has taken place in public opinion as to the necessity of medical training for missionaries, which is now generally regarded as a necessity. The President's address was followed by a speech from Professor Alexander Macalister, which paid tribute to the Principal, an old Cambridge pupil. He expressed entire confidence in the training given at the College, which enables a missionary to render simple medical aid to the natives. After an appreciative speech from Dr. M. A. Stein, the Central Asian explorer, Mr. McAdam Eccles moved a vote of thanks to the Chairman and speakers, which was seconded by Dr. Price.

After June 24, 1909, the Royal Sanitary Institute and Parkes Museum will be removed to 90 Buckingham Palace Road, London, S.W.

The annual dinner of the British Balneological and Climatological Society will be held this year on Friday, June 11, at the Piccadilly Restaurant, London, W., when the President, Dr. Ernest Solly, of Harrogate, will occupy the chair.

The summer dinner of the West African Medical Staff will take place on Monday, June 21, at the New Gaiety Restaurant, Strand, London, W. Members of the staff who desire to be present are requested to communicate immediately with Dr. Prout, C.M.G., 78 Rodney Street, Liverpool.

Convocation of the University of Oxford on Tuesday last approved unanimously two decrees accepting from Dr. Theodore Williams £2,500 to establish two scholarships in physiology, and £2,500 to establish two scholarships in human anatomy. The Vice-Chancellor proposed the decrees and expressed the gratitude of the University to Dr. Williams for his liberal benefactions.

The Lord Mayor has received a letter from the Treasurer of St. Bartholomew's Hospital pointing out that the Governors feel very seriously the heavy burden of the rates to which the buildings used in connection with the Charity are subjected. The Governors ask whether, in the event of their promoting a Bill in Parliament for their exemption from rates, they may receive the support of the Corporation in their efforts to obtain relief.

The administrators of the Red Cross Fund, established by the Dowager Empress Marie Feodorovna of Russia to provide prizes for inventions designed to relieve the sufferings of the wounded in time of war, are now in Paris for the purpose of deciding the subjects for the next competition in 1912. Her Majesty's gift of about £10,000 was made in 1902 on the occasion of the seventh International Red Cross Conference at St. Petersburg, and the interest is spent upon the fund's quinquennial prizes. The first prizes, three in number, of the approximate value of £600 each, were awarded in 1907 at the International Red Cross Conference in London, for inventions offering the quickest and surest methods of discovering and transporting the wounded. The second competition will take place in 1912 at the ninth International Conference to be held at Washington.

